# Optimized scaling of translational factors in oncology: from xenografts to RECIST

**DOI:** 10.1007/s00280-022-04458-8

**Published:** 2022-08-03

**Authors:** Marcus Baaz, Tim Cardilin, Floriane Lignet, Mats Jirstrand

**Affiliations:** 1 Fraunhofer-Chalmers Research Centre for Industrial Mathematics, Chalmers Science Park, 41288 Gothenburg, Sweden; 2 Department of Mathematical Sciences, Chalmers University of Technology, University of Gothenburg, Gothenburg, Sweden; 3 Merck Healthcare KGaA, Darmstadt, Germany

**Keywords:** Translational research, Combination therapy, Oncology, Mathematical modeling, Nonlinear mixed effects

## Abstract

**Purpose:**

Tumor growth inhibition (TGI) models are regularly used to quantify the PK–PD relationship between drug concentration and in vivo efficacy in oncology. These models are typically calibrated with data from xenograft mice and before being used for clinical predictions, translational methods have to be applied. Currently, such methods are commonly based on replacing model components or scaling of model parameters. However, difficulties remain in how to accurately account for inter-species differences. Therefore, more research must be done before xenograft data can fully be utilized to predict clinical response.

**Method:**

To contribute to this research, we have calibrated TGI models to xenograft data for three drug combinations using the nonlinear mixed effects framework. The models were translated by replacing mice exposure with human exposure and used to make predictions of clinical response. Furthermore, in search of a better way of translating these models, we estimated an optimal way of scaling model parameters given the available clinical data.

**Results:**

The predictions were compared with clinical data and we found that clinical efficacy was overestimated. The estimated optimal scaling factors were similar to a standard allometric scaling exponent of − 0.25.

**Conclusions:**

We believe that given more data, our methodology could contribute to increasing the translational capabilities of TGI models. More specifically, an appropriate translational method could be developed for drugs with the same mechanism of action, which would allow for all preclinical data to be leveraged for new drugs of the same class. This would ensure that fewer clinically inefficacious drugs are tested in clinical trials.

**Supplementary Information:**

The online version contains supplementary material available at 10.1007/s00280-022-04458-8.

## Introduction

A major problem in the drug development process in oncology is translating results from preclinical studies to a clinical setting [1, 2]. Clinical efficacy is frequently overpredicted, which means that test compounds showing promising preclinical results fail when they enter clinical trials [3, 4]. This is one of the main reasons for the high attrition rates seen for anticancer drugs [5]. However, there exists a correlation between preclinical efficacy, estimated from studies using either patient-derived xenografts (PDXs) or traditional xenografts based on cell lines, and clinical efficacy [6–8]. This shows the potential of using xenograft mice for testing compounds, in particular PDXs as they represent the human disease condition better [9], but also highlights the need for further translational research.

Combination therapies have come to play a leading role in anticancer treatment during the last decades [10]. The strengths of this type of treatment are, *e.g.*, synergistic effects between the drugs and slower onset of resistance [11, 12]. However, giving two drugs concomitantly leads to complex pharmacokinetic (PK) as well as pharmacodynamic (PD) interactions that need to be analyzed [13]. Moreover, these effects can differ between species, making translational efforts even more challenging [14].

Mathematical modeling is a powerful tool for drug development and, in particular, for evaluating combination therapies [15]. Typically, a tumor growth inhibition (TGI) model is developed and calibrated to xenograft tumor volume data [16–18], and then used to investigate the efficacy of alternative treatments scenarios such as different drug doses or treatment schedules [19]. However, inter-species differences have to be accounted for to make clinical predictions [20]. The translational methods that are currently used can primarily be divided into two categories: replacement of model components, *e.g.*, growth rate parameter, exposure, or even the entire PK model; and scaling of model parameters [21, 22]. However, these methods are often insufficient to accurately translate the relationship between drug dose or concentration and in vivo efficacy due to the physiological differences between tumor xenograft in mice and cancer progression in human [23]. A potential contributing factor is also inadequate experimental design [24]. Therefore, additional model-based translational approaches are needed to make full use of preclinical data and minimize drug attrition rates.

In this paper, we calibrate preclinical TGI models using xenograft data from the literature for three drug combinations. We then replace mice PK with human PK, accounting for differences in protein binding, and formulate a mathematical optimization problem to find how to best scale the PD rate parameters to describe published clinical data. We hypothesize that the optimal scaling factors could be drug/cancer type specific and could thus be used to leverage all preclinical data when developing new drug combinations for the same cancer type and with the same drug mechanisms of action. Finally, we compare the optimal scaling factor with the standard allometric scaling factor for rate parameters.

## Methods

### Data

#### Preclinical data

We analyzed PDX data for combination therapies for which we were also able to find clinical data in the literature. The PDXs had either cutaneous melanoma (CM) or colorectal cancer (CRC) and data for combinations of binimetinib/encorafenib (CM), binimetinib/ribociclib (CM), and cetuximab/encorafenib (CRC) were taken from Gao et al., 2015 [7]. Data for vehicle groups of the two cancer types and single agent data were also extracted. All-time series were cut at day 60 to better reflect a typical xenograft study. Exposure data for encorafenib and ribociclib in mice were extracted from the same publication, whereas data for the other two drugs were gathered from other sources [25, 26]. Treatment schedules and sample size of each treatment group can be found in the Supplementary Information (Table S1).

Anticancer drugs can have different efficacy depending on the specific cancer cells mutations the patient has [27]. We have, therefore, stratified the data into BRAF-mutants, NRAS-mutants, and all other mutants. In the binimetinib/ribociclib combination group, there were five CM PDXs that had a mutation in the BRAF gene and five that had a mutation in the NRAS gene. There were 13 BRAF mutants and nine NRAS mutants in all other CM treatment groups. Among the CRC PXDs, there were only six BRAF mutants and a single NRAS mutant.

### Clinical data

In clinical oncology studies, patient response is categorized using the RECIST criteria. The sum of the longest diameters for all target lesions (SLD) is measured at the start of treatment (baseline) and at subsequent checkups. Each patient is categorized based on their best response using four response categories: Complete Response (CR), Partial Response (PR), Progressive Disease (PD), and Stable Disease (SD) [28].

Clinical RECIST data were obtained from ClinicalTrials.gov. Data for the following treatment groups were available: binimetinib (NRAS/BRAF, CM) [29, 30], binimetinib/ribociclib (NRAS, CM) [31], encorafenib (BRAF, CM) [32], binimetinib/encorafenib (BRAF, CM) [32], cetuximab (CRC) [33], and encorafenib/cetuximab (CRC, BRAF) [34]. All drugs were given orally, except for cetuximab, which was given intravenously. Treatment schedule, sample size, checkup time, response rate, cancer type, and mutations for each clinical trial can be found in the Supplementary Information (Table S1). For more information regarding each study, the reader is referred to the corresponding article.

### Preclinical modeling

#### Exposure to anticancer drugs

Daily unbound average concentration, ${C}_{avg,u}$, was used to describe the exposure to all drugs except binimetinib for which unbound maximum concentration, ${C}_{max,u}$ was instead used, as maximal concentration has been shown to correlate better with clinical efficacy than overall exposure for this particular compound [26]. The unbound concentrations were computed by first estimating the total average or maximum concentration, ${C}_{avg,tot}\, \mathrm{or}\, {C}_{max,tot}$, and then adjusting for in vitro mean unbound protein fraction in mice, ${f}_{u, Mouse} (17)$, according to

1 $$C_{avg,u} = C_{avg,tot} \cdot f_{u, Mouse} ,\,C_{max,u} = C_{max,tot} \cdot f_{u, Mouse} ,$$

${f}_{u, Mouse}$ for each drug was extracted from the literature [25, 35–37]. Compartmental models were fitted to the extracted exposure data of encorafenib and ribociclib. One-compartment models were sufficient to describe the PK data of both compounds. For cetuximab, we used a one-compartment model from the literature [25]. These three models were used to estimate ${C}_{avg,tot}$ of encorafenib, ribociclib, and cetuximab in the TGI model. Due to lack of an adequate PK model for binimetinib, the ${C}_{max,tot}$ value was gathered from the literature [26]. Total and unbound exposure for each drug and treatment schedule are summarized in Table 1. A detailed description of how these values were derived is available in the Supplementary Information.

**Table 1:** Preclinical and clinical drug exposure

Drug	Dose schedule	Total exposure	${{\varvec{f}}}_{{\varvec{u}}}$	Unbound exposure
Preclinical				
Cetuximab	20 mg/kg 2.q.w.	3235 $\mathrm{\mu g}/\mathrm{mL}$	1	3235 $\mathrm{\mu g}/\mathrm{mL}$
	20 mg/kg q.2.w.	893 $\mathrm{\mu g}/\mathrm{mL}$		893 $\mathrm{\mu g}/\mathrm{mL}$
Encorafenib	20 mg/kg b.i.d.	28 $\mathrm{\mu g}/\mathrm{mL}$	0.042	1.2 $\mathrm{\mu g}/\mathrm{mL}$
	20 mg/kg q.d.	14 $\mathrm{\mu g}/\mathrm{mL}$		0.6 $\mathrm{\mu g}/\mathrm{mL}$
Ribociclib	250 mg/kg q.d.	16 $\mathrm{\mu g}/\mathrm{mL}$	0.2	3.2 $\mathrm{\mu g}/\mathrm{mL}$
Binimetinib	10 mg/kg b.i.d.	1.2 $\mathrm{\mu g}/\mathrm{mL}$	0.015	0.02 $\mathrm{\mu g}/\mathrm{mL}$
Clinical				
Cetuximab [34, 38]	400/250 mg/m2 q.w.	3236 $\mathrm{\mu g}/\mathrm{mL}$	1	3236 $\mathrm{\mu g}/\mathrm{mL}$
Encorafenib [32]	300 mg q.d.	6.60 $\mathrm{\mu g}/\mathrm{mL}$	0.14	0.92 $\mathrm{\mu g}/\mathrm{mL}$
Encorafenib [32, 35]	450 mg q.d.	8.25 $\mathrm{\mu g}/\mathrm{mL}$	0.14	1.15 $\mathrm{\mu g}/\mathrm{mL}$
Ribociclib [31]	200 mg q.d.	4.00 $\mathrm{\mu g}/\mathrm{mL}$	0.3	1.2 $\mathrm{\mu g}/\mathrm{mL}$
Binimetinib[32]	45 mg b.i.d.	0.60 $\mathrm{\mu g}/\mathrm{mL}$	0.03	0.02 $\mathrm{\mu g}/\mathrm{mL}$

Specification of both total and unbound preclinical and clinical exposure for each drug and treatment schedule

### Tumor growth inhibition model

**Fig. 1:**
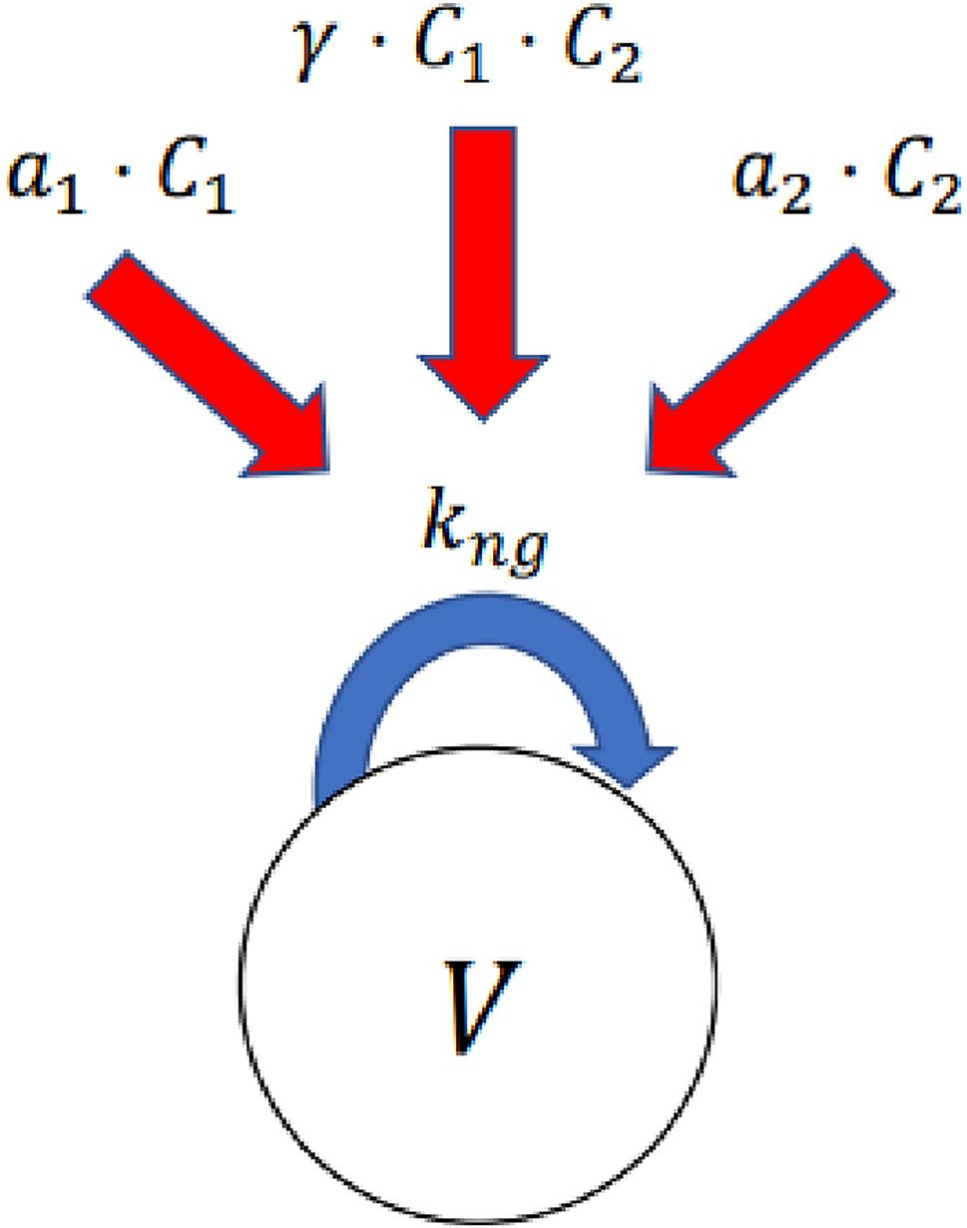
A schematic representation of the TGI model for two drugs. *V* denotes the volume of the proliferating cells and ${k}_{ng}$ the net tumor growth rate constant before start of treatment. ${C}_{i}$ and ${a}_{i}$ are the unbound concentration and potency of drug *i*, respectively. There is also a possible interaction term between the two drugs, denoted by ${\gamma }_{i,j}.$

To quantify the preclinical anticancer efficacy of each drug and drug combination, a one-compartment TGI model was calibrated to each tumor type. The choice of this relatively simple model was made to balance model complexity with the amount of available data. In the model, all tumor cells are assumed to be proliferating and located in a single compartment. A schematic representation of the model is shown in Fig. 1.

Turnover of tumors cells exposed to drug *i* as single agent is described by the following differential equation,

2 $$\frac{dV}{{dt}} = \left( {k_{ng}^{ } - a_{i}^{ } \cdot C_{i}^{ } } \right)V\left( t \right), V\left( 0 \right) = V_{0} ,$$

where *V* is the volume of tumor cells, ${V}_{0}$ the initial tumor volume, ${k}_{ng}$ the net tumor growth rate constant, ${a}_{i}$ the potency of drug *i*, and ${C}_{i}$ average or maximum unbound drug concentration. T

When two drugs, *i* and *j*, are given in combination, the turnover is instead described by,

3 $$\frac{dV}{{dt}} = \left( {k_{ng}^{ } - a_{i}^{ } \cdot C_{i}^{ } - a_{j}^{ } \cdot C_{j}^{ } - \gamma_{ i,j}^{ } C_{i}^{ } C_{j}^{ } } \right)V\left( t \right),$$

where ${\gamma }_{i,j}$ is included to describe a potential synergistic or antagonistic effect between the drugs [9].

Mathematical modeling and parameter estimation were performed using an NLME framework (more details are found in Computational Methods). One TGI model for each cancer type was fitted to the data and log-normal between-subject variability (BSV) was accounted for on the parameters ${k}_{ng}$ and ${V}_{0}$ in both models and on the potency parameter of binimetinib, ${a}_{Bini}$, in the CM model. No correlation between random effects was assumed and a proportional observation error was used in the model based on residual analysis. We also investigated if there was a significant difference between parameter estimates if treatment groups were stratified in BRAF-mutants, NRAS-mutants, and others.

### Clinical modeling

#### Translational

To predict clinical response, translational methods were applied to the preclinical TGI models. Initially, we only replaced mouse exposure with human exposure, after accounting for differences in protein binding [20, 21]. For each drug, reported $AU{C}_{tot}$ or ${C}_{max,tot}$ values were taken from the clinical study if available, or otherwise values from similar studies. The exposure was then adjusted by in vitro mean unbound protein fraction in humans, ${f}_{u, Human}$ [6, 24, 35, 36]. Total and unbound exposures for each drug and treatment schedule are summarized in Table 1. A detailed description of how values were derived is available in the Supplementary Information.

### Clinical predictions

We used our translated preclinical TGI models to predict the proportion of patients in each RECIST category. To do this, two important aspects first had to be considered. First, the RECIST criteria are based on SLD, whereas predictions from the models are on volumes. Therefore, we converted the volume predictions to SLD by assuming either spherical or ellipsoid tumors [39]. In the ellipsoid case, prolate ellipsoids were assumed as well as that tumor growth or shrinkage only occurs along the longest radius. This leads to the volumetric change being the same as the change in SLD between two time points. For the spherical case, the volumetric change has to be greater than the SLD change to achieve CR/PR or PD [28, 39, 40]. Both assumptions of spherical and ellipsoid tumors were evaluated in this paper.

Second, only the best response, which can occur at any checkup, for each patient is reported in the clinical studies. Therefore, we made the simplifying assumption that the best response occurred at the first evaluation, *i.e.,* at week 6 or 8, and we called this time $T$. We subsequently investigated how the predictions were affected if a different $T$ was chosen.

To make the predictions, we used the translated preclinical model (formed by the preclinical tumor model combined with the human PK) to generate 1000 studies with the same number of individuals as in the original study. The time evolution of tumor volume of each individual was simulated and converted to SLD. After that, the percentage change between baseline and week $T$ was calculated, using the following equation,

5 $${\Delta }SLD = 100 \cdot \frac{{SLD_{T} - SLD_{0} }}{{SLD_{0} }}.$$

**Fig. 2:**
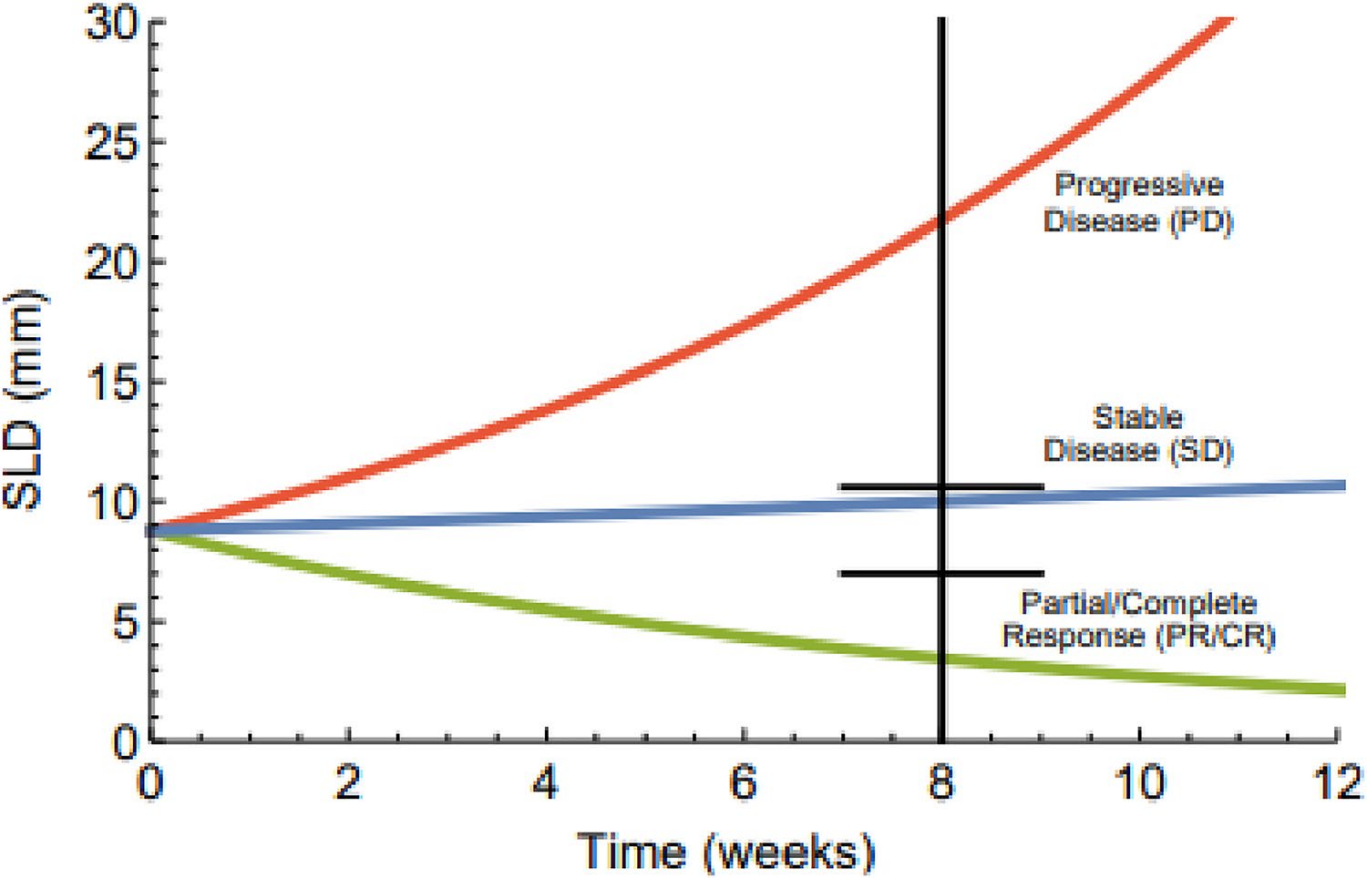
An illustration of how clinical predictions are performed. The color of green, blue, and red denotes classification into PR/CR, SD, or PD, respectively. The change in SLD between baseline and week 8 (black, vertical line) is compared to classify each individual

A patient is classified as CR&PR if$\Delta SLD\le -30$, as PD if $\Delta SLD\ge 20$, and as SD if $-30\le\Delta SLD\le 20$ [28]. This process of generating and categorizing individuals is illustrated in Fig. 2.

Each individual’s $\Delta SLD$ was compared with the RECIST thresholds and thus, the proportion of patients in each RECIST category was estimated for each study. Subsequently, mean and 95% confidence interval (PCI*)* of each RECIST category was calculated. We considered a prediction to be adequately good if the PCI covers the clinical data observation.

### Optimization

After making our predictions with the translated models we wanted to investigate how the parameters in the model should be scaled to describe the clinical data better. The parameters that we focused on were the PD rate parameters, ${k}_{ng}$, ${a}_{i}$, and ${\gamma }_{i,j}$. We allowed the scaling of these parameters to be different and denoted the optimal scaling factors for them by A, B, and C, respectively. The optimal scaling factors were introduced to the model using the following expressions and were found by formulating and solving an optimization problem.

$$k_{ng}^{H} = A \cdot k_{ng}^{M}$$

$$a_{i}^{H} = B \cdot a_{i }^{M}$$

6 $$\gamma_{ }^{H} = C \cdot \gamma_{ }^{M}$$

Here the superscript, $M$, denotes that the parameter is estimated from PDX data and, $H$, that the parameter is scaled for human predictions.

To formulate the optimization problem, we denoted the clinically observed and predicted percentage of patients in RECIST category *i* for treatment group *j* by ${y}_{ij}$ and ${y}_{ij}^{*}$*,* respectively. Furthermore, ${y}_{ij}^{*}$ is a function of the scaling factors $x=(A, B, C).$ A least-squares problem was formulated to find *x* such that the difference between ${y}_{ji}$ and ${y}_{ij}^{*}$ is minimized for all *i* and *j*. Mathematically this is described by the equation,

7 $$f\left( x \right) = \mathop \sum \limits_{i,j} \left( {y_{ij}^{*} \left( x \right) - y_{ij} } \right)^{2} .^{ }$$

However, this objective function can lead to optimal solutions where some RECIST categories are not adequately predicted, which is compensated by very accurate predictions of other categories. Thus, to improve the predictions, on a study level, we penalized the solution for each RECIST category in *y* that was not covered by the PCI*.* This promotes solutions with as many adequate predictions as possible and was done by introducing the following penalty term,

8 $$\lambda \mathop \sum \limits_{i,j} g_{ij} \left( x \right) = 0,$$

where $\lambda$ is a penalty constant and,

9 $$g_{ij} \left( x \right) = \left\{ {\begin{array}{*{20}l} {0\,{\text{ if}}\,y_{ij} \, \in PCI_{j} } \\ {1\,{\text{ if}}\,y_{ij} \, \notin PCI_{j} .} \\ \end{array} } \right.$$

Combining this penalty term with Eq. 7 results in the following equation,

10 $$L\left( x \right) = f\left( x \right) + \lambda \mathop \sum \limits_{i,j} g_{j} \left( x \right).$$

The optimization problem was formulated as,

11 $${\text{minimize}} \; L\left( x \right),\,{\text{subject to }} - \infty < x \le 0.$$

The optimization procedure was validated by first synthesizing data with known optimal scaling factors and then re-estimating these known factors. To give an idea of the uncertainty of the estimates, a non-parametric bootstrap was performed to calculate RSE % of each optimal scaling factor.

### Allometric scaling

The heart rate of organisms has been shown to be proportional to the body weight of the organism raised to power of −0.25 [41]. This is the underlying rationale for some to propose that parameters associated with tumor growth can also be allometric scaling with exponent −0.25 [42]. Standard values of the body weight of a human and a mouse are assumed to be 70 kg and 20 g, respectively, which results in a scaling factor of approximately 0.13. We compared this scaling factor with the optimal scaling factors we found through our optimization procedure.

### Computational methods

Mathematical modeling and parameter estimation were performed using an NLME modeling approach based on the first-order conditional estimation (FOCE) method. The computational framework used was developed at the Fraunhofer-Chalmers Research Centre for Industrial Mathematics (Gothenburg, Sweden) [43]. The preclinical TGI models were simultaneously fitted to tumor volume data from all treatment groups of the same cancer type. The models were introduced based on the precision of estimated parameters, individual fits, empirical Bayes estimates (EBEs), Akaike information criterion (AIC), and visual predictive checks (VPC). We used Simulated Annealing and set $\lambda$ to 1000 to solve the optimization problem. Mathematica was used to create all figures and to perform all computations.

## Results

### Preclinical modeling

#### Pharmacodynamics

The preclinical TGI models were able to describe the xenograft data adequately. Two outliers were removed from the cetuximab (CRC) and encorafenib (CM) treatments groups. Examples of individual fits and parameter estimates are shown in Fig. 3 and Table 2, respectively. Fits to the entire dataset can be found in the supplementary information (Figs. S4–S13). VPCs can be found in the Supplementary Information (Figs. S1 and S2). All model parameters were estimated with acceptable precision.

**Fig. 3:**
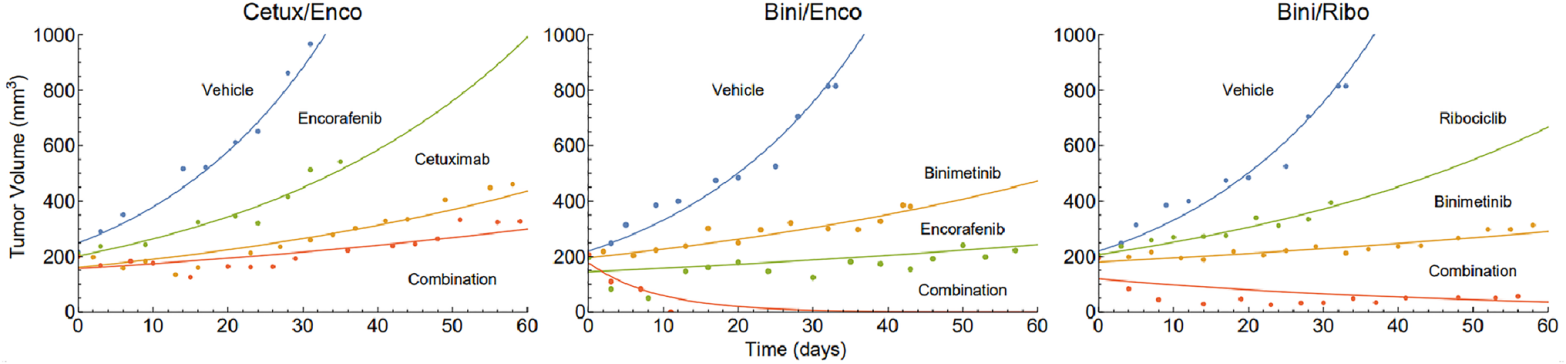
Tumor volume versus time for one individual per treatment group and drug combination. Continuous lines are model predictions and dots experimental observations

**Table 2:** Parameter estimates

	Parameter	Unit	Estimate (RSE %)	BSV (RSE %)
Colorectal cancer	${k}_{ng}$	$1/\mathrm{day}$	0.05 (6)	71 (12)
	${V}_{o}$	${\mathrm{mm}}^{3}$	235 (2)	24 (12)
	${a}_{Cetu}$	$\mathrm{mL}/(\mathrm{\mu g}\cdot \mathrm{day}$)	$9.4\cdot$ 10^–6^ (9)	
	${a}_{Enco}$	$\mathrm{mL}/(\mathrm{\mu g}\cdot \mathrm{day}$)	0 (-)	
	${\gamma }_{Cetu,Enco}$	${\mathrm{mL}}^{2}/{\left(\upmu {\mathrm{g}}^{2}\cdot \mathrm{day}\right)}$	$2.6\cdot$ 10^–5^ (9)	
Cutaneous melanoma	${k}_{ng}$	$1/\mathrm{day}$	0.06 (6)	53 (15)
	${V}_{o}$	${\mathrm{mm}}^{3}$	200 (2)	22 (14)
	${a}_{Enco}$	$\mathrm{mL}/(\mathrm{\mu g}\cdot \mathrm{day}$)	0.12 (10)	
	${a}_{Ribo}$	$\mathrm{mL}/(\mathrm{\mu g}\cdot \mathrm{day}$)	0.013 (9)	
	${a}_{Bini}$	$\mathrm{mL}/(\mathrm{\mu g}\cdot \mathrm{day}$)	1.7 (19)	61 (35)

Estimated PD parameters after fitting the two TGI models to the xenograft tumor volume data

*RSE* relative standard error

Mutations did not significantly affect the net tumor growth rate constant or initial tumor volume. However, a significant difference in potency of encorafenib and binimetinib was found between different mutations. BRAF mutated PDXs responded considerably better to encorafenib than other PDXs. The potency parameter of encorafenib for BRAF-mutated CM PDXs was estimated to be 0.12 $mL/(\mu g\cdot day)$, whereas no effect could be estimated for non BRAF-mutated CM PDXs. Since there were only six BRAF mutated CRC PDXs among 43, no significant encorafenib potency could be estimated for this sub-group either. Only the model describing the combination of cetuximab with encorafenib required an interaction term, which was estimated with good precision to 2.6 $\cdot$ 10^–5^ $m{L}^{2}/(\mu {g}^{2}\cdot day)$.

### Clinical modeling

#### Clinical predictions

**Fig. 4:**
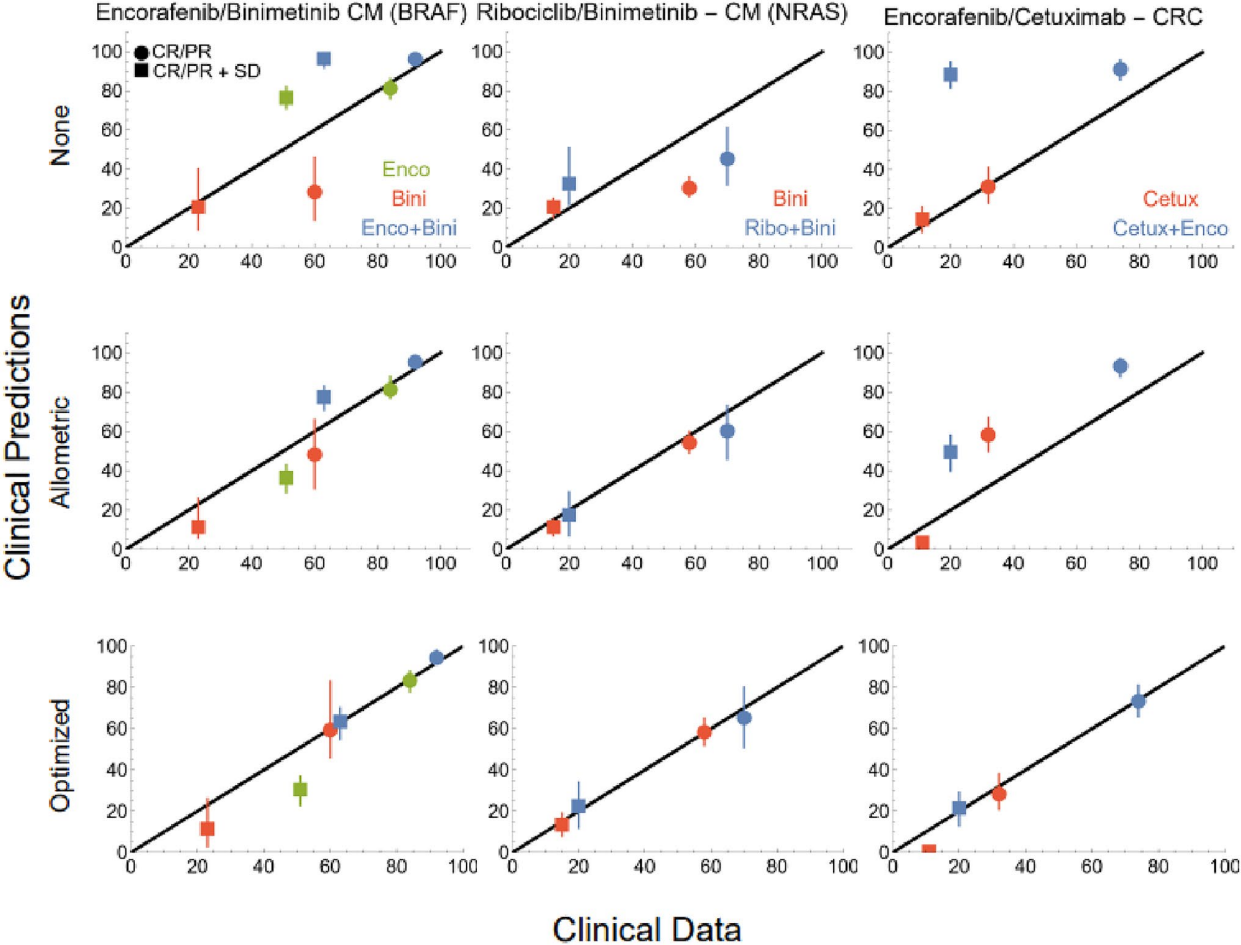
(Row 1 and 2) Clinical predictions plotted against clinical data for all drug combinations and using both replacement of PK and allometric scaling. (Row 3) Illustration of how well the translated model, using the optimal scaling factors could describe the clinical data. Color denotes treatment group and circles represent the response categories CR/PR and squares CR/PR + SD

The preclinical TGI models were translated and clinical predictions were made for each treatment group where clinical data was available. As the predictions using ellipsoid tumors were in better agreement with the clinical data for the timeframe we used, we only present these predictions. A plot showing how the predictions are affected by the choice of $T$ can be found in the Supplementary Information (Figure S3). The predictions, including predictions using allometric scaling, plotted against the clinical data for each treatment group are shown in Fig. 4, and in Table S4 in the Supplementary Information.

### Optimization

The result of the validation procedure of the optimization method is found in the Supplementary Information (Table S2). The optimization problem, formulated in, Eq. 11, was solved separately for four monotherapy treatment groups. The optimal scaling factors (A, B) for each drug, along with RSE of each estimate and clinical predictions, can be found in the Supplementary Information (Table S3).

The optimization problem was also solved for the drug combinations. The preclinical cetuximab/encorafenib TGI model was the only combination with an interaction term and, therefore, three scaling factors. Results from the optimization for each drug combination are shown in Table 3. How well the translated models, using these optimal scaling factors, were able to describe the clinical data are shown in the last row of Fig. 4 and in the Supplementary Information (Table S4).

**Table 3:** Optimization results

Treatment (cancer)	A (RSE %)	B (RSE %)	C (RSE %)
Monotherapies			
Enco (CM-BRAF)	0.20 (9)	0.20 (6)	–
Bini (CM-BRAF)	0.17 (3)	0.23 (3)	–
Bini (CM-NRAS)	0.14 (5)	0.18 (7)	–
Cetux (CRC)	0.85 (16)	0.78 (16)	–
Combinations			
Bini/Enco	0.07 (19)	0.09 (17)	–
Bini/Ribo	0.12 (10)	0.14(11)	–
Cetux/Enco	0.21 (15)	0.09 (45)	0.13 (10)

Optimal scaling factors, with RSE, for each drug and drug combination

## Discussion

### Preclinical modeling

#### Exposure

The use of unbound concentrations is vital to account for differences in protein binding of species. This is especially the case for highly bound drugs, as a slight difference in protein binding can significantly affect the active drug concentration at the target site [44]. However, a limitation of using unbound fractions, estimated in vitro*,* is that it can be misleading as it does not necessarily describe the free concentration at the target site accurately [45]. Despite this, we do still believe that the approach we have used is the most appropriate for the data available to us.

In the final preclinical PD model we used a single value $({C}_{avg,u}$ or ${C}_{max,u}$) to represent the exposure of the different drugs. These were chosen based on information from the literature what correlated best with clinical efficacy. We also tested using the simulated PK profiles of both encorafenib and binimetinib to drive the PD model, but this did not alter the fit.

Since clinical tumor measurements are typically performed quite infrequently (*e.g.,* once every 8^th^ week) we do not believe that a more dynamical PK model than the one we used would significantly improve the model predictions.

### Pharmacodynamics

#### Pharmacodynamic model

The PK-PD relationship between drug concentration and in vivo efficacy is commonly described by linear expressions, with the possible inclusion of an interaction term [46, 47]. Since the drugs we investigated were only tested at one dose level, the preclinical TGI models used need to match this lack of richness in the experimental data. The choice of a relatively simple model was made after testing other models as well, *e.g.,* the Simeoni model (13), but not being able to estimate all parameters with sufficient precision given the available data.

All treatment groups with PDXs created from the same cancer type were fitted simultaneously and the models were able to describe the xenograft tumor volume data adequately. All model parameters were estimated with acceptable precision. The models were able to describe the data on both an individual level as well as on a population level, as can, *e.g.,* be seen in the individual fits and VPCs, respectively.

### Analysis of mutations

Our predictions of the potency of each drug with regards to mutation are in agreement with the result from previous studies. BRAF-inhibitors, to which encorafenib belongs, are efficacious against BRAF-mutated CM but not against NRAS-mutated CM, which is what the model also predicted [48]. Moreover, the CRC PDXs seemed to be insensitive to encorafenib, given as monotherapy, however, the analysis is biased by the limited number of CRC BRAF-mutants in the dataset used.

### Clinical predictions

#### Predictions versus data

Clinical predictions were made for all three drug combinations using the translated preclinical TGI models. With mice PK replaced with human PK, the model tended to overpredict the drug efficacy. This overprediction can, to some extent, be explained by the fact that the drugs were only tested on one preclinical dose level and, therefore, the model potency functions had to be extrapolated. Moreover, encorafenib was found to only have an effect when it was given as a combination to CRC PDXs, and thus the need to extrapolate the interaction term further explains the overprediction of the cetuximab and encorafenib combination. Having preclinical data for multiple dose levels would have been optimal, to minimize prediction errors coming from extrapolation.

Another aspect that must be considered is BSV and inter-study variability (ISV). BSV was included in on the tumor growth rate parameter to account for significant variability observed in the preclinical vehicle group. However, as there are no vehicle group in clinical studies it could be hard to quantify how much of the clinical variability comes from differences in tumor growth and drug sensitivity. ISV can be quite large when studies are conducted at different time periods, locations, or by different scientists [38]. As the data we have used have been taken from different literature sources, we expected significant inter-study variability.

Furthermore, the predictions are also affected by the choice of model. We used semi-mechanistic models, and it would be interesting to investigate how much the predictions could be improved if more mechanistic models instead were used. To better mimic the human disease condition tumor regrowth or the appearance of new lesions could for example be included in the model. However, the model complexity is limited by shortcomings of PDXs such as they *e.g.,* only have one tumor lesion.

To evaluate what type of model is more suitable for this type of translational research, the cost, in terms of model complexity and biological knowledge, could be compared with the improvement in predictions. To perform such an evaluation, richer datasets, including different dose levels both in monotherapy and in combination may also be needed.

### Assumptions

We made two assumptions in our clinical prediction: how to convert volumetric model predictions to diameters and when the best response of the patients occurred. Evidence has previously been put forward that assuming an ellipsoid tumor geometry is a better prognostic indicator of overall survival than a spherical one [39, 40]. Overall survival is thought to be correlated to response rates [49] and thus, it follows that ellipsoids should be more suitable for classifying patient response. Our findings further showed the superiority of assuming ellipsoid tumors, as these predictions were in better agreement with the clinical data.

Furthermore, we assumed that the best response of all patients occurred at the first checkup, *i.e.,* after 6 or 8 weeks from the start of treatment. We based this assumption on two studies that found that change in tumor volume at week 8 [50] and after two cycles of chemotherapy [51] correlated significantly with overall survival. We also analyzed how this assumption affects the predictions by varying the time of best response, which we call $T$. The number of patients that are classified as SD will shrink towards zero as $T$ is increased. Moreover, the predictions from our approach will converge towards those using the Tumor-Static Concentration concept [52], as $T$ becomes sufficiently large (see Figure S3 in the Supplementary Information). Figure S3 could be used to investigate what the best choice of *T* is. However, in our approach we chose to fix *T* and instead focus the investigation on how the predictions were affected by scaling the model parameters.

Other researchers who also have predicted clinical response rates from PDX data have made similar time frame assumptions. For example, Wong et al*.* [6] compared preclinical TGI after three weeks with clinical response rates and Lindauer et al*.* [22] simulated tumor volumes and categorized the population at 0, 1, 3, and 6 months after treatment started. Pierrillas et al*.* proposed a different approach for comparing preclinical and clinical efficacy based on allometric scaling of the time frame [53]. They compared preclinical model predictions with human PD responses after 42 days of treatment, which compares well with our assumption of 6–8 weeks.

### Optimal scaling factors

#### Optimization problem

The optimization problem was first solved for single agents and then for all three drug combinations. RSE of the optimal scaling factors was also calculated through a bootstrap procedure. The results from single agent optimization showed that the optimal scaling factors were all estimated with acceptable precision and were greater than the allometric scaling factor.

The optimal scaling factors for the cetuximab/encorafenib combination ranged from 0.09 to 0.21, which is significantly larger than the range of the factors of the other two combinations. However, as it was the only combination that showed a significant interaction effect, this difference in range is somewhat hard to interpret. It could indicate the need for different scaling approaches than the one we used when modeling combination therapies with significant potency interaction effects.

### Comparison with allometric scaling

The allometric scaling relationship applies to endogenous mouse and human tumors, and some consideration has to be made to PDXs. In PDXs, human tumors are growing in mouse microenvironments and, therefore, there may exist a scaling factors that better captures the differences between PDXs and humans.

The optimal scaling factors that we found were quite close to the standard allometric scaling factor, giving some validity to the idea of scaling TGI models in this way. However, as can be seen in Fig. 4, standard allometric scaling was not sufficient to predict clinical response for all the combinations under study. Thus, showing that in order for the predictions from the translated preclinical TGI models to fit the clinical data for the combination therapies under study, the PD rate parameters had to, in general, be scaled down more than the allometric theory suggests. This demonstrates the need for new and improved scaling techniques, which might be found by testing our optimized scaling method on a more extensive dataset. A more suitable scaling factor, possibly specific to cancer type and mechanism of action of the drug(s), could then be proposed. This factor would be used to describe the differences between humans and xenograft mice more accurately and would allow all preclinical data to be leveraged early in the drug development process. This should help in reducing the risks of bringing clinically inefficacious drugs to the clinical development stage.

## Conclusions

The predicted clinical efficacy of the three drug combinations was generally overestimated from the translated preclinical TGI models. More informative preclinical data in combination with a more complex model could potentially improve the predictions.

We developed a methodology for finding an appropriate scaling factor for TGI models. The methodology was applied to the drug combinations and we found that the optimal scaling factors were generally smaller than what allometric scaling suggests. However, more drug combinations have to be analyzed before a general factor can be proposed. To continue exploring and improving the translation capability of semi-mechanistic model, more data is required.

## Supplementary Information

Below is the link to the electronic supplementary material.

Supplementary file1 (DOCX 1293 KB)

## Data Availability

The preclinical data can freely be accessed from Gao et al*.* [1]. All supporting Mathematica code is available from the corresponding author on reasonable request.
