# Supplementary material for: Optimized scaling of translational factors in oncology: from xenografts to RECIST
Source: Cancer Chemother Pharmacol. 2022 Aug 3;90(3):239–50. doi: 10.1007/s00280-022-04458-8 (PMC9402719; doi:10.1007/s00280-022-04458-8)
Supplement: Supplementary file 1 — Supplementary file1 (DOCX 1293 KB) [file 280_2022_4458_MOESM1_ESM.docx]

# Supplementary Information

# Text

## Clinical Trials

The COLOMBUS study (NCT01909453) was a randomized phase 3 study that compared the efficacy of encorafenib (450 mg oral q.d.) in combination with binimetinib (45 mg b.i.d.) and encorafenib (300 mg oral q.d.) monotherapy in patients with BRAF-mutant melanoma [1]. For the 24 first weeks, tumors were evaluated every 8^th^ week. Response rates for the combination group (N=192) were 63% CR/PR and 92% CR/PR + SD, and in the monotherapy (N=192), the numbers were 51% CR/PR and 84% CR/PR + SD.

The efficacy of binimetinib (45 mg b.i.d.) for patients (N=269) with NRAS-mutant melanoma was investigated in the randomized phase 3 study NEMO (NCT01763164) [2]. Tumors were assessed every 6^th^ week for the first 25 weeks and response rates were CR/PR 15% and CR/PR + SD 58%. The efficacy of binimetinib (45 mg b.i.d.) in combination with ribociclib (200 mg q.d.) for patients (N=40) with NRAS-mutant melanoma was also investigated in a 2-month phase II study (NCT01781572) [3]. Response rates in this study were CR/PR 20% and CR/PR + SD 70%. Moreover, in a different study, binimetinib (45 mg b.i.d.) was evaluated for both NRAS (N=30) and BRAF (N=45) mutated melanomas (NCT01320085) [4]. As the NRAS group consisted of fewer patients than the NEMO study, we only used the data for the BRAF mutation. Checkups were conducted at the end of each second cycle and response rates were CR/PR 23% and CR/PR+SD 60%.

Cetuximab (400 mg/250 mg per m^2^ q.w.) was investigated as a monotherapy (N=111) for patients with CRC (no specific mutation) [5]. It was also investigated in combination with encorafenib (300 mg q.d.) for patients (N=113) with BRAF-mutated CRC (NCT02928224) [6]. In both studies, patients were evaluated every 6^th^ week for the first 24 weeks. Response rates for monotherapy were CR/PR 11% and CR/PR+SD 32%. For the combination therapy, the numbers were CR/PR 20% and CR/PR+SD 74%.

## Preclinical Exposure

A one-compartment model with absorption, taken from the literature, is used to estimate $AUC_{tot}$ for the 20 mg/kg cetuximab 2qw and q2w treatment groups [7]. By simulating the model, we arrive at estimates of 3235 $\mu g/mL$ and 893 $\mu g/mL$, respectively. No significant protein binding is expected for cetuximab since it is a humanized monoclonal IgG1 antibody, *i.e.,* $f_{u,Mouse}$ is approximately 1 [8].

One-compartment models without absorption are sufficient to describe the PK data for encorafenib and ribociclib. From these models $AUC_{tot}$ is calculated for encorafenib to be 14 $\mu g\cdot h/mL$ and 28 $\mu g\cdot h/mL$ for the 20 mg q.d. and b.i.d treatment groups, respectively, and 16 $\mu g\cdot h/mL$ for 250 mg/kg ribociclib. The *in vitro* mean unbound protein fraction of the active metabolite of encorafenib in rodents has been reported to be high, with $f_{u,Rat}$ being 0.042 [9]. We assume that protein binding in rats and mice is similar and that it is the same for the active metabolite as for encorafenib. $f_{u,Mouse}$ for ribociclib is reported to be 0.2 [10].

$C_{max,tot}$for a single i.v. dose of 30 mg/kg binimetinib is reported in the literature to be 6.8 $\mu g/mL$ and bioavailability to 54% [11]. Since the preclinical dose is given orally and only 10 mg/kg, we estimate $C_{max,tot}$ in the preclinical study as $0.54\cdot\frac{1}{3}\cdot6.8 \frac{\mu g}{mL}=$ 1.2 $\mu g/mL$. $f_{u,Mouse}$ of binimetinib is above 0.04 for many preclinical species, but the specific value for mice was not found in the literature [12]. However, cobimetinib, a drug with similar mechanisms of action, is slightly more bound to proteins in mice than in humans [13]. Therefore, we assumed a similar relationship for binimetinib and that $f_{u,Mouse}$of mice is 0.015, which is slightly above the human value, 0.03. Furthermore, assuming this degree of protein binding leads to $AUC_{u}$ that is similar in both humans and mice.

## Clinical Exposure

$AUC_{0-6h,tot}$ of cetuximab is reported in the clinical trial to be 841 $\mu g\cdot h/mL$ and another study reports daily $AUC_{tot}$ to be approximately 3020 $\mu g\cdot h/mL$ [6,14]. These values line up very well with the model predictions for 20 mg/kg cetuximab 2qw (preclinical monotherapy group), as $AUC_{0-6h,tot}$ is estimated to 791 $\mu g\cdot h/mL$ and daily $AUC_{tot}$ to 3236 $\mu g\cdot h/mL$. Therefore, we assume that the total exposure is the same in the preclinical monotherapy group as in the clinical monotherapy and combination therapy groups. This means that the exposure in the clinical combination group is approximately 3.6 times larger than in the preclinical combination group. Since no significant protein binding is expected, we assume that the total and unbound exposure are the same [8].

$AUC_{tot}$ for a 300 mg encorafenib dose is reported to be 6.6 $\mu g\cdot h/mL$ [1]. To obtain the $AUC_{tot}$ for the 450 mg q.d. clinical group$,$the reported $AUC_{tot}$ for 300 mg is scaled with a factor of 1.3, as $AUC_{tot}$ increase less than dose proportionally [9]. $f_{u,Human}$ is reported to be 0.14, which gives an $AUC_{u}$ of 0.92 and 1.15 for the 300 mg and 450 mg doses, respectively [9].

Steady state $AUC_{tot}$ of 200 mg ribociclib is reported to be approximately 4 $\mu g\cdot h/mL$ in the clinical efficacy study [3]. $f_{u,Human}$ of ribociclib is 0.3, which gives an $AUC_{u}$ of 1.2 $\mu g\cdot h/mL$ [10].

Steady state $C_{max,tot}$ of binimetinib, is reported to be approximately 0.7$\mu g/mL$ in the COLUMBUS trial [1]. $f_{u,Human}$ of binimetinib is approximately 0.03 and therefore $C_{max,u}$ is estimated to be 0.02 $\mu g/mL$ [12].

# Tables

**Table S1** Preclinical and Clinical Data Summary

| Treatment | NCT | Sample size | Cancer (Mutation) | Checkups every | CR/PR % | SD % |
| --- | --- | --- | --- | --- | --- | --- |
| Preclinical | | | | | | |
| Vehicle | - | 45 | CRC | - | - | - |
| 20 mg/kg b.i.d. encorafenib | - | 43 | CRC | - | - | - |
| 20 mg/kg 2.q.w. cetuximab | - | 43 | CRC | - | - | - |
| 20 mg/kg b.i.d encorafenib +  20 mg/kg q.2.w. cetuximab | - | 42 | CRC | - | - | - |
| Vehicle | - | 45 | CM | - | - | - |
| 10 mg/kg b.i.d. binimetinib | - | 33 | CM | - | - | - |
| 20 mg/kg q.d. encorafenib | - | 33 | CM | - | - | - |
| 250 mg/kg q.d. ribociclib | - | 33 | CM | - | - | - |
| 10 mg/kg b.i.d. binimetinib +  20 mg/kg q.d. encorafenib | - | 33 | CM | - | - | - |
| 10 mg/kg b.i.d. binimetinib +  250 mg/kg q.d. ribociclib | - | 18 | CM | - | - | - |
| Clinical | | | | | | |
| 45 mg binimetinib b.i.d [2] | 01763164 | 269 | CM (NRAS) | 6th week | 15 | 58 |
| 45 mg binimetinib b.i.d [4] | 01320085 | 45 | CM (BRAF) | 8th week | 23 | 60 |
| 300 mg q.d. encorafenib [1] | 01909453 | 192 | CM (BRAF) | 8th week | 51 | 84 |
| 450 mg q.d. encorafenib +  45 binimetinib b.i.d [1] | 01909453 | 192 | CM (BRAF) | 8th week | 63 | 92 |
| 45 mg binimetinib b.i.d +  200 mg ribociclib q.d. [3] | 01781572 | 40 | CM (NRAS) | 2 months* | 20 | 70 |
| 400 mg/250 mg per m^2^ q.w. cetuximab [5] | - | 111 | CRC (**) | 6th week | 11 | 32 |
| 400 mg/250 mg per m^2^ q.w. cetuximab + 300 mg encorafenib q.d. [6] | 02928224 | 113 | CRC (BRAF) | 6th week | 20 | 74 |

Specification of treatment schedule, sample size, cancer type, mutation, checkup interval, and response rate of each preclinical and clinical treatment group. q.2.w.: once every 2nd week, 2.q.w.: twice weekly, q.d.: once daily, b.i.d.: twice daily. * Checkup after two months ** No specific mutation.

**Table S2** Validation of Optimization Result

| **Parameter difference** | **Single Agent** | **1 Combination** | **3 Combination** |
| --- | --- | --- | --- |
| **0-10 %** | 88 % | 95 % | 97 % |
| **10 > %** | 12 % | 5 % | 3 % |

Validation of the optimization result. The optimization problem was solved for generated data, with known optimal scaling factors. The table shows the difference between the true value and optimal value found through optimization.

**Table S3** Single Agent Optimization

|  | | |
| --- | --- | --- |
| **CM (BRAF)** | **Enco** | |
|  | Clinical | Predicted |
| **Optimization** |  | |
| CR/PR | 51 | 51 (45 - 58) % |
| CR/PR+SD | 84 | 84 (79 - 89) % |
| **CM (BRAF)** | **Bini** | |
|  | Clinical | Predicted |
| **Optimization** |  | |
| CR+PR | 23 | 23 (9 - 37) % |
| CR+PR+SD | 60 | 60 (43-80) % |
| **CM (NRAS)** | **Bini** | |
|  | Clinical | Predicted |
| **Optimization** |  | |
| CR+PR | 15 | 15 (12 - 20) % |
| CR+PR+SD | 58 | 58 (53 - 65) % |
| **CRC** | **Cetux** | |
|  | Clinical | Predicted |
| **Optimization** |  | |
| CR+PR | 11 | 11 ( 5- 17) % |
| CR+PR+SD | 32 | 33 (24 - 41) % |

Optimal scaling factors and RSE% for monotherapy groups along with how well the model, using these factors, could describe the clinical data. Groups are marked green if the 95% confidence interval of the prediction covers the clinical data and red otherwise.

**Table S4** Combination Therapy Clinical Predictions and Optimized Model Descriptions

| Response | Clinical | 1 | 2 | 3 |
| --- | --- | --- | --- | --- |
| Cutaneous Melanoma (BRAF) | | | | |
| Encorafenib | | | | |
| CR/PR | 51 % | 76 (71-82) % | 36 (29-43) % | 30 (23-37) % |
| CR/PR+SD | 84 % | 82 (76-86) % | 82 (77-88) % | 84 (78-88) % |
| Binimetinib | | | | |
| CR/PR | 23 % | 20 (9-40) % | 11 ( 3-26) % | 11 ( 3-26) % |
| CR/PR+SD | 60 % | 29 (14-46) % | 49 (31-66) % | 60 (46-83) % |
| Encorafenib + Binimetinib | | | | |
| CR/PR | 63 % | 96 (92-97) % | 77 (71-83) % | 63 (55-70) % |
| CR/PR+SD | 92 % | 97 (93-99) % | 96 (92-98) % | 95 (92-98) % |
| Cutaneous Melanoma (NRAS) | | | | |
| Binimetinib | | | | |
| CR/PR | 15 % | 20 (15-25) % | 11 ( 7-15) % | 13 ( 8-19) % |
| CR/PR+SD | 58 % | 31 (26-36) % | 55 (49-60) % | 59 (52-65) % |
| Ribociclib + Binimetinib | | | | |
| CR/PR | 20 % | 32 (22-51) % | 17 ( 7-29) % | 22 (12-34) % |
| CR/PR+SD | 70 % | 46 (32-61) % | 61 (46-73) % | 66 (51-80) % |
| Colorectal Cancer | | | | |
| Cetuximab | | | | |
| CR/PR | 11 % | 14 (8-21) % | 3 (0-6)% | 0 (0-3) % |
| CR/PR+SD | 32 % | 32 (23-41) % | 59 (45-69) % | 29 (21–38) % |
| Cetuximab + Encorafenib | | | | |
| CR/PR | 20 % | 88 (82-95) % | 49 (40-58) % | 21 (13-29) % |
| CR/PR+SD | 74 % | 92 (86-96) % | 94 (88-97) % | 74 (66-81) % |

Prediction of clinical response rate for (1) replacement of PK, (2) using standard allometric scaling factors, (3) how well the model, using optimized scaling factors could describe the clinical data. Groups are marked green if the 95% confidence interval of the prediction covers the clinical data and red otherwise.

# Figures


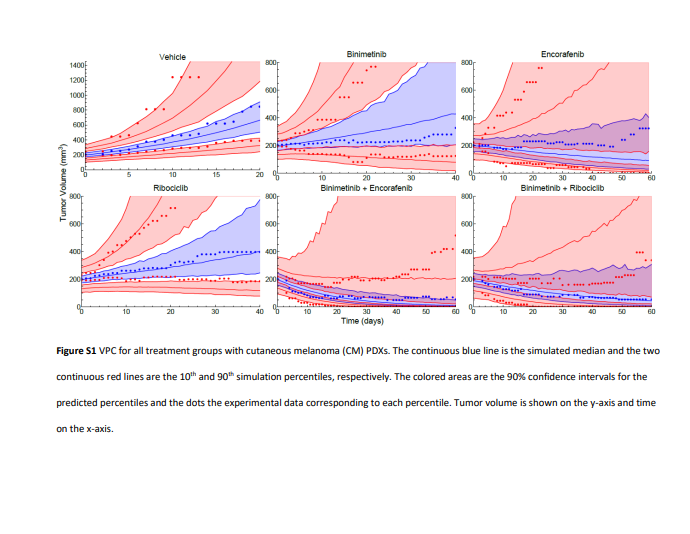


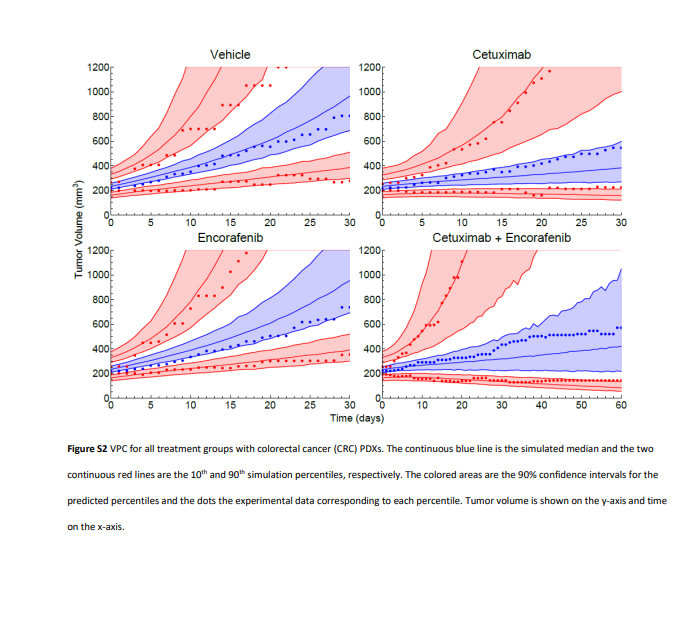


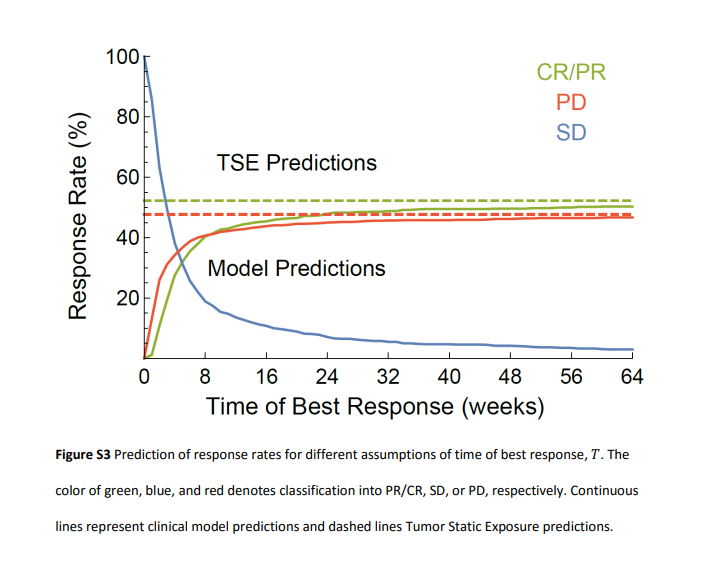


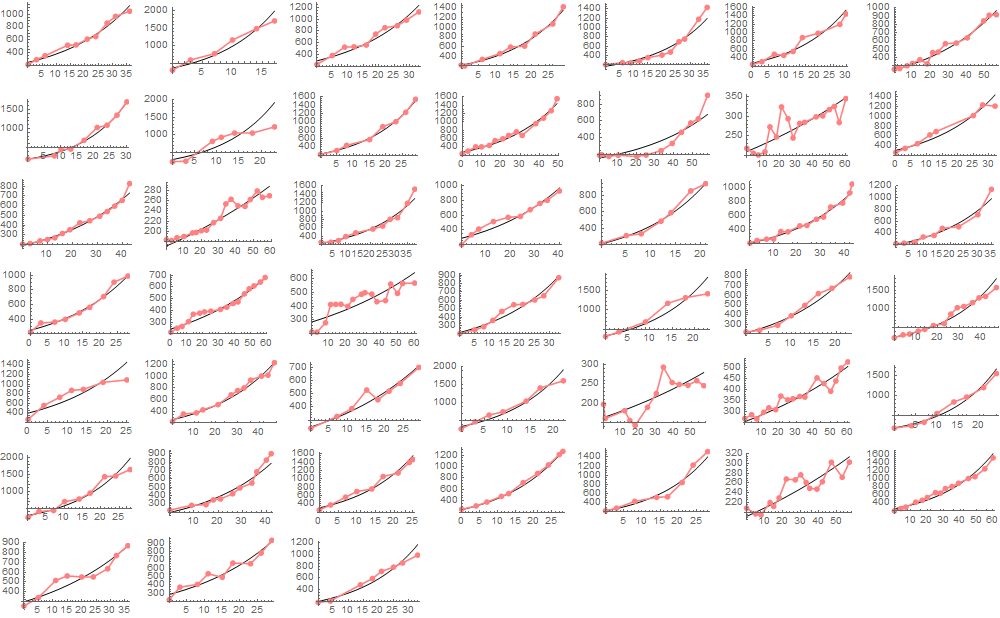


**Figure S4** Model fits to each individual in the CRC vehicle group. Red dots are experimental data and black lines model predictions. X and Y-axes represent time (days) and tumor volume ($mm^{3}$).


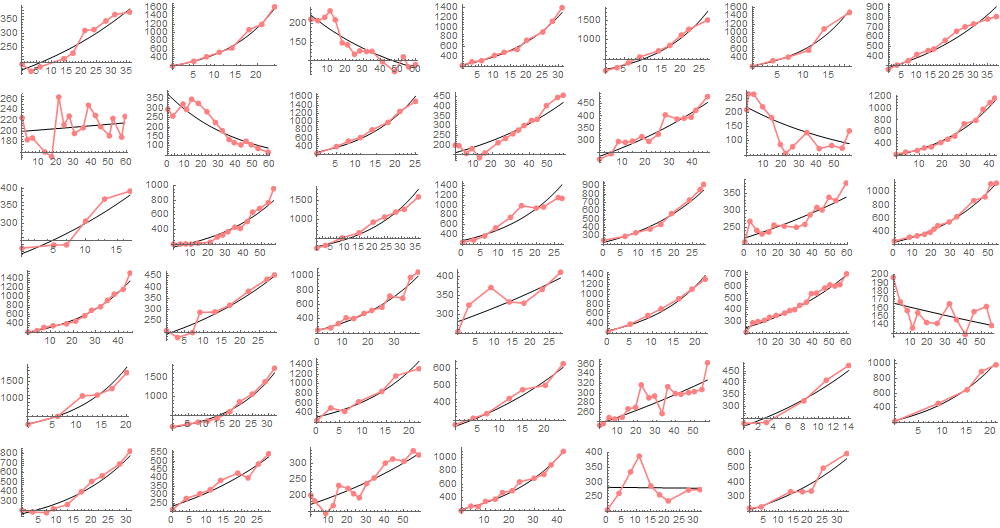


**Figure S5** Model fits to each individual in the CRC cetuximab group. Red dots are experimental data and black lines model predictions. X and Y-axes represent time (days) and tumor volume ($mm^{3}$).


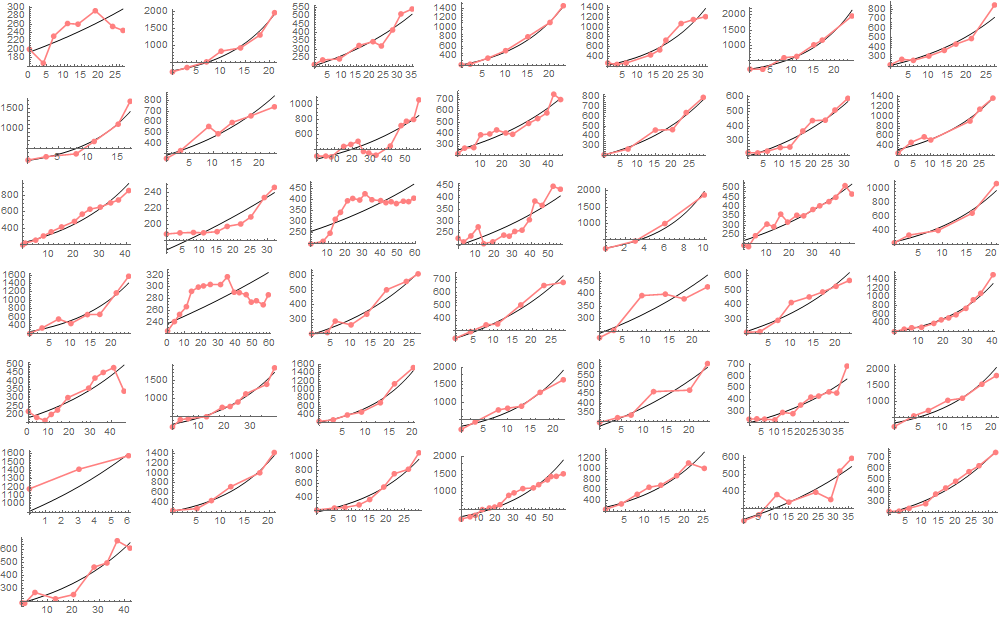


**Figure S6** Model fits to each individual in the CRC encorafenib group. Red dots are experimental data and black lines model predictions. X and Y-axes represent time (days) and tumor volume ($mm^{3}$).


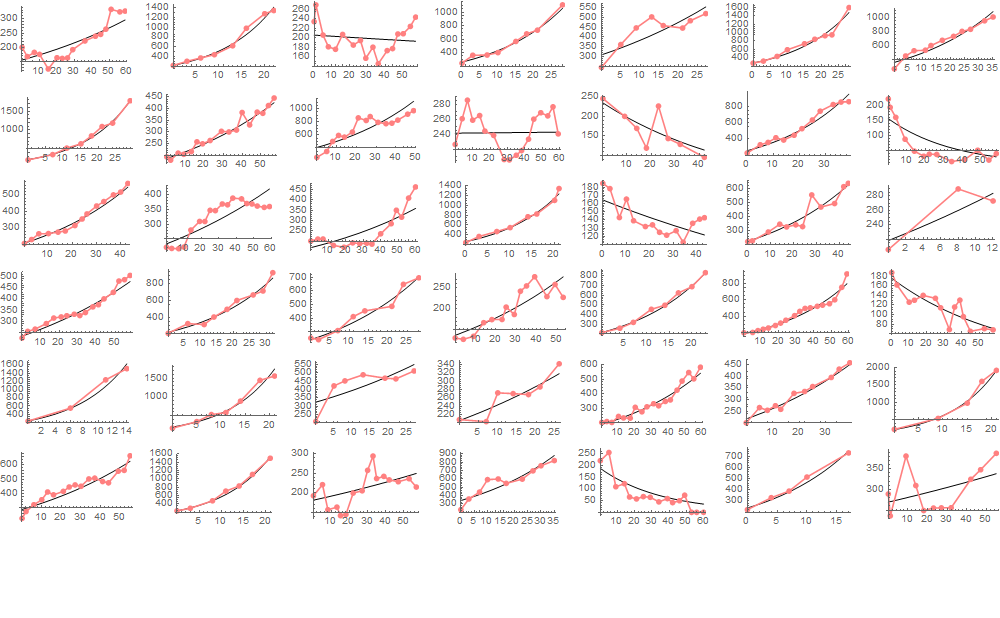


**Figure S7** Model fits to each individual in the CRC cetuximab /encorafenib combination group. Red dots are experimental data and black lines model predictions. X and Y-axes represent time (days) and tumor volume ($mm^{3}$).


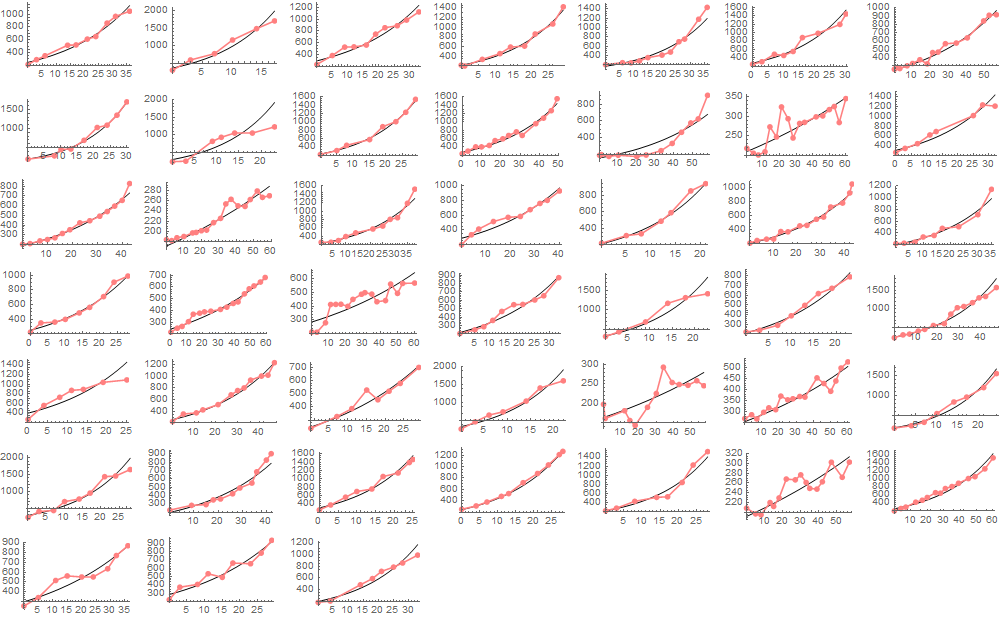


**Figure S8** Model fits to each individual in the CM vehicle group. Red dots are experimental data and black lines model predictions. X and Y-axes represent time (days) and tumor volume ($mm^{3}$).


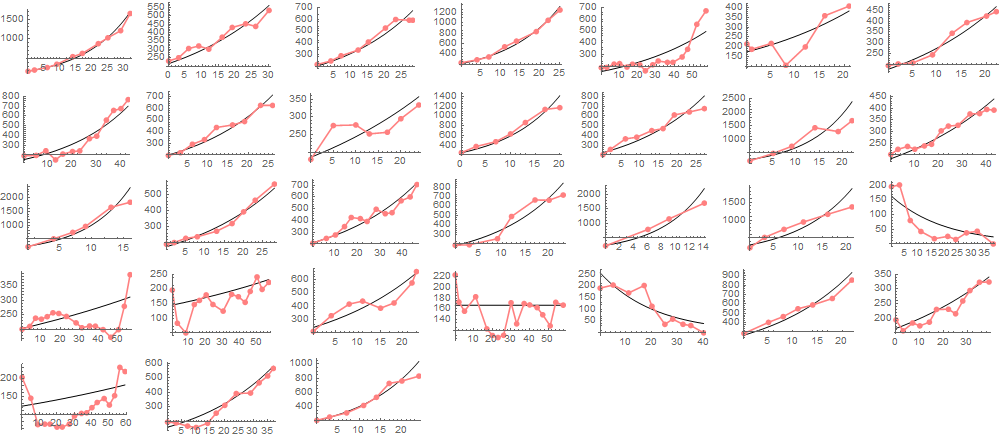


**Figure S9** Model fits to each individual in the CM encorafenib group. Red dots are experimental data and black lines model predictions. X and Y-axes represent time (days) and tumor volume ($mm^{3}$).


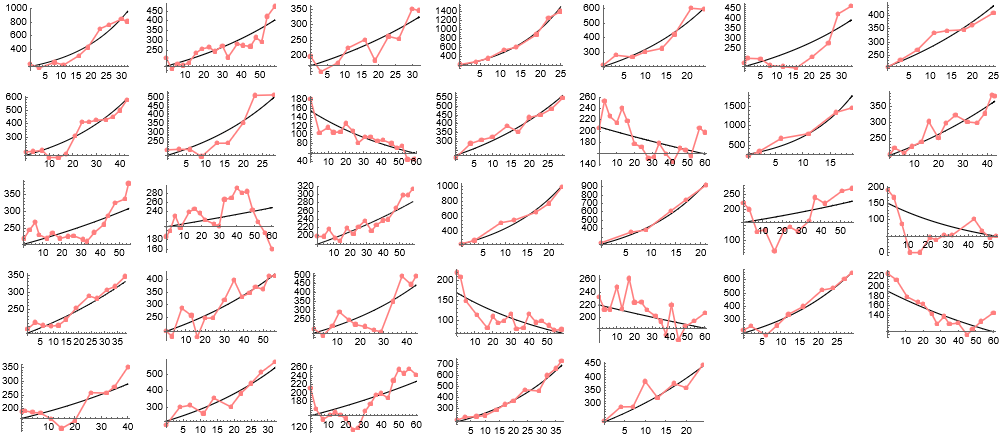


**Figure S10** Model fits to each individual in the CM encorafenib group. Red dots are experimental data and black lines model predictions. X and Y-axes represent time (days) and tumor volume ($mm^{3}$).


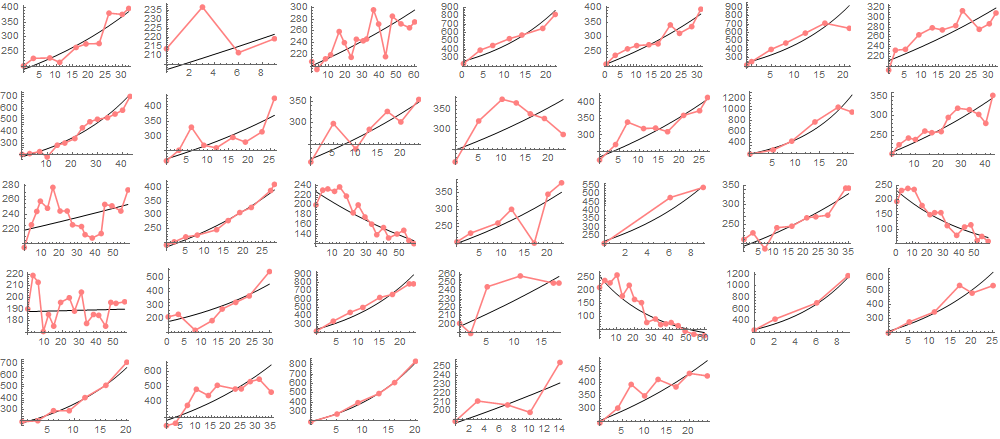


**Figure S11** Model fits to each individual in the CM ribociclib group. Red dots are experimental data and black lines model predictions. X and Y-axes represent time (days) and tumor volume ($mm^{3}$).


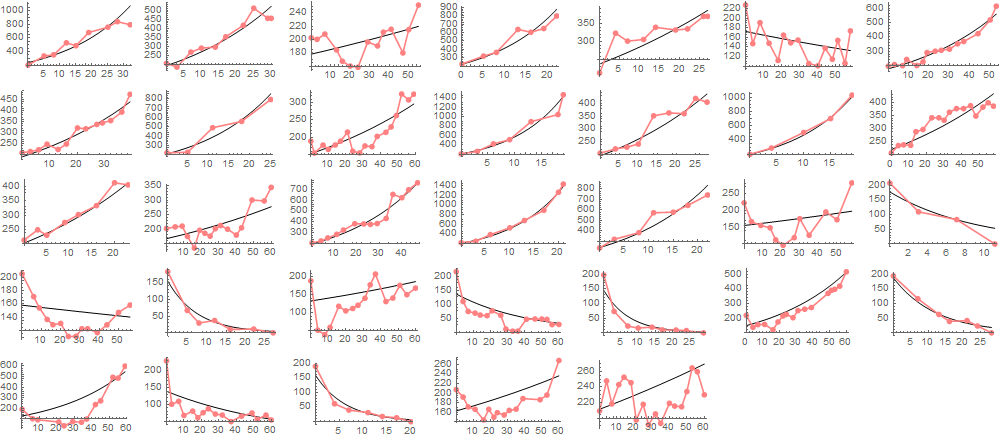


**Figure S12** Model fits to each individual in the CM encorafenib/binimetinib combination group. Red dots are experimental data and black lines model predictions. X and Y-axes represent time (days) and tumor volume ($mm^{3}$).


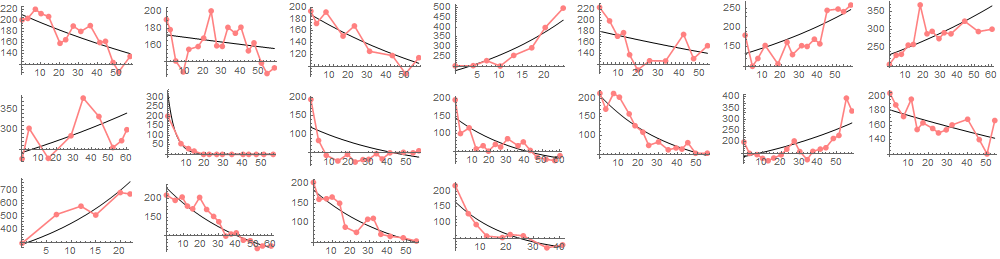


**Figure S13** Model fits to each individual in the CM ribocliclib/binimetinib combination group. Red dots are experimental data and black lines model predictions. X and Y-axes represent time (days) and tumor volume ($mm^{3}$).

# References

[1] Dummer R, Ascierto PA, Gogas HJ, Arance A, Mandala M, Liszkay G, et al. Encorafenib plus binimetinib versus vemurafenib or encorafenib in patients with BRAF-mutant melanoma (COLUMBUS): a multicentre, open-label, randomised phase 3 trial. Lancet Oncol 2018;19:603–15. https://doi.org/10.1016/S1470-2045(18)30142-6.

[2] Dummer R, Schadendorf D, Ascierto PA, Arance A, Dutriaux C, Giacomo AMD, et al. Binimetinib versus dacarbazine in patients with advanced NRAS-mutant melanoma (NEMO): a multicentre, open-label, randomised, phase 3 trial. Lancet Oncol 2017;18:435–45. https://doi.org/10.1016/S1470-2045(17)30180-8.

[3] Sosman JA, Kittaneh M, Lolkema MPJK, Postow MA, Schwartz G, Franklin C, et al. A phase 1b/2 study of LEE011 in combination with binimetinib (MEK162) in patients with NRAS-mutant melanoma: Early encouraging clinical activity. J Clin Oncol 2014;32:9009–9009. https://doi.org/10.1200/jco.2014.32.15_suppl.9009.

[4] Ascierto PA, Schadendorf D, Berking C, Agarwala SS, van Herpen CM, Queirolo P, et al. MEK162 for patients with advanced melanoma harbouring NRAS or Val600 BRAF mutations: a non-randomised, open-label phase 2 study. Lancet Oncol 2013;14:249–56. https://doi.org/10.1016/S1470-2045(13)70024-X.

[5] Cunningham D, Humblet Y, Siena S, Khayat D, Bleiberg H, Santoro A, et al. Cetuximab Monotherapy and Cetuximab plus Irinotecan in Irinotecan-Refractory Metastatic Colorectal Cancer. N Engl J Med 2004;351:337–45. https://doi.org/10.1056/NEJMoa033025.

[6] Kopetz S, Grothey A, Yaeger R, Cutsem EV, Desai J, Yoshino T, et al. Encorafenib, Binimetinib, and Cetuximab in BRAF V600E–Mutated Colorectal Cancer. N Engl J Med 2019;381:1632–43. https://doi.org/10.1056/NEJMoa1908075.

[7] Luo FR, Yang Z, Dong H, Camuso A, McGlinchey K, Fager K, et al. Prediction of Active Drug Plasma Concentrations Achieved in Cancer Patients by Pharmacodynamic Biomarkers Identified from the Geo Human Colon Carcinoma Xenograft Model. Clin Cancer Res 2005;11:5558–65. https://doi.org/10.1158/1078-0432.CCR-05-0368.

[8] Luo FR, Yang Z, Dong H, Camuso A, McGlinchey K, Fager K, et al. Correlation of pharmacokinetics with the antitumor activity of Cetuximab in nude mice bearing the GEO human colon carcinoma xenograft. Cancer Chemother Pharmacol 2005;56:455–64. https://doi.org/10.1007/s00280-005-1022-3.

[9] European Medicines Agency. Assessment report Braftovi Procedure No. EMEA/H/C/004580/0000 2018.

[10] European Medicines Agency. Assessment report Kisqali Procedure No. EMEA/H/C/004213/0000 2017.

[11] Center for Drug Evaluation and Research. Multi-Disciplinary Review and Evaluation NDA 210498 MEKTOVI^TM^ 2015.

[12] European Medicines Agency. Assessment report Mektovi Procedure No. EMEA/H/C/004579/0000 2018.

[13] Center for Drug Evaluation and Research. Pharmacology Review of Cotellic n.d.

[14] European Medicines Agency. Scientific Discussion on Erbitux 2004.
